# New-onset seizures after cranioplasty—a different view on a putatively frequently observed phenomenon

**DOI:** 10.1007/s00701-021-04720-7

**Published:** 2021-02-01

**Authors:** Dorian Hirschmann, Beate Kranawetter, Matthias Tomschik, Jonathan Wais, Fabian Winter, Josa M. Frischer, Matthias Millesi, Johannes Herta, Karl Roessler, Christian Dorfer

**Affiliations:** Department of Neurosurgery, Medical University Vienna, Waehringer Guertel 18-20, 1090 Vienna, Austria

**Keywords:** Cranioplasty, Seizures, Risk factors, Follow-up

## Abstract

**Background:**

New-onset seizures after cranioplasty (NOSAC) are reported to be a frequent complication of cranioplasty (CP) after decompressive hemicraniectomy (DHC). There are considerable differences in the incidence of NOSAC and contradictory data about presumed risk factors in the literature. We suggest NOSAC to be a consequence of patients’ initial condition which led to DHC, rather than a complication of subsequent CP. We conducted a retrospective analysis to verify our hypothesis.

**Methods:**

The medical records of all patients ≥ 18 years who underwent CP between 2002 and 2017 at our institution were evaluated including incidence of seizures, time of seizure onset, and presumed risk factors. Indication for DHC, type of implant used, timing of CP, patient age, presence of a ventriculoperitoneal shunt (VP shunt), and postoperative complications were compared between patients with and without NOSAC.

**Results:**

A total of 302 patients underwent CP between 2002 and 2017, 276 of whom were included in the outcome analysis and the incidence of NOSAC was 23.2%. Although time between DHC and CP differed significantly between DHC indication groups, time between DHC and seizure onset did not differ, suggesting the occurrence of seizures to be independent of the procedure of CP. Time of follow-up was the only factor associated with the occurrence of NOSAC.

**Conclusion:**

New-onset seizures may be a consequence of the initial condition leading to DHC rather than of CP itself. Time of follow-up seems to play a major role in detection of new-onset seizures.

## Introduction

According to numerous reports given in the literature, new-onset seizures are a common complication of cranioplasty (CP). While some authors advocate that new-onset seizures after cranioplasty (NOSAC) are a consequence of procedural complications, such as hemorrhage, hydrocephalus, and wound healing disorder [13, 17, 22], others focus on the identification of other risk factors [16, 18–21]. According to these mainly retrospective analyses, risk factors include patient age at CP, time between decompressive craniectomy (DHC) and CP, presence of a ventriculoperitoneal shunt (VP shunt) at time of CP, type of implant used, and indication for DHC. Even hypertension and diabetes mellitus have been reported to be risk factors for NOSAC [19]. However, data seem to be inconsistent, and in some cases contradictory. Among numerous studies, the incidence of NOSAC ranges widely (from 4.1 to 30.3%) even between comparable patient cohorts [16] [9, 11, 17, 19]. Moreover, definite risk factors could not be identified [18]. Although seizures generally may occur after neurosurgical operations, NOSAC have to be analyzed separately and in a more differentiated way due to the fact that the initial pathologies leading to DHC—and consequently CP—commonly cause seizures themselves [3, 8, 12, 15]. We suggest the phenomenon of NOSAC to be mainly an effect of the initial condition of patients undergoing DHC and subsequent CP, and that the differences in their observed frequency were not only caused by massive bias but to be very much dependent on time of follow-up. We conducted a retrospective study to evaluate our hypothesis on the basis of our own data.

## Methods

We evaluated a consecutive series of 302 patients who underwent cranioplasty (CP) between 2002 and 2017 at our institution focusing on the incidence of NOSAC during postoperative follow-up. The following data were extracted from the medical records: indication for DHC, timing of CP, pre- and postoperative seizure status, and postoperative complications leading to reoperation. Furthermore, the time of the very first seizure in relation to DHC and CP was documented for each patient. Seizure status was determined according to reporting of the occurrence of epileptic seizures within the medical record. EEG confirmation of seizures was not a prerequisite in this study. Patients with a history of epilepsy prior to the initial disease leading to DHC were screened but excluded from the outcome analysis. For all remaining patients, seizure status after CP was determined according to seizure occurrence only after CP and seizures between DHC and CP. In addition, we evaluated whether only a single seizure or multiple seizures occurred after CP. Patients with a minimum age of 18 years were included.

To analyze presumed risk factors for new-onset seizures, the following variables were compared between patients with and without seizures after CP: indication for DHC, timing of CP, patient age, and incidence of postoperative complications (such as hemorrhage, epi- and subdural fluid collection, and bone graft infection). To evaluate a potential association of time of follow-up with the incidence of NOSAC as a source of bias, time of follow-up was also compared between the two patient groups.

According to our institution’s policies, antiepileptic drugs are not administered routinely for prophylaxis before, during, or after CP in patients without a history of seizures. Patients who suffer from a single seizure at any time are routinely given 2000 mg levetiracetam daily for at least 6 months.

### Statistical analysis

For comparison between groups, chi-squared test, Mann-Whitney *U* test, Kruskal-Wallis test, and logistic regression were used. The Kaplan-Meier plot was used to calculate actuarial rates of new-onset seizures and differences between groups were compared with log-rank test. *P* values < 0.05 were considered significant. Statistical analysis was performed with IBM SPSS Statistics 24. This study has been approved by the ethics committee of the authors’ institution (EK 2244/2017).

## Results

Between 2002 and 2017, a total of 302 patients underwent cranioplasty at the authors’ institution. Seven of the 302 patients had already suffered from epileptic seizures prior to DHC and thus were excluded from the outcome analysis. In one additional patient who was excluded from the outcome analysis, the seizure status prior to CP could not be determined due to an insufficient medical record. Another 18 patients who were younger than 18 years at the time of CP were excluded. Consequently, 276 patients remained for the outcome analysis.

The median age at cranioplasty was 49 years (18–81 years) and 141 patients (51.1%) were male. In 258 patients (93.5%), an autologous bone graft was used for cranioplasty and a synthetic graft in 18 patients (6.5%). Indications for DHC were evenly distributed including subarachnoid hemorrhage (30.1%), ischemic stroke (27.2%), intraparenchymal hemorrhage (21.4%), and traumatic brain injury (19.2%). Only a minority of procedures were performed for other indications including brain abscess, tumor, and fulminant encephalitis with edema (2.2%) (Table 1). DHC was performed on the left side in 38.4% (106/276), on the right in 57.2% (158/276), and bilaterally in 4.3% (12/276). The overall median follow-up was 29.5 months (0–211 months). In 87.7% of patients, a minimum follow-up time of 1 month was available for analysis.

**Table 1:** Indications for craniectomy and rates of new-onset seizures

Indication for craniectomy (*n* = 276)	Patients, *n* (%)	Patients with new-onset seizures after cranioplasty (%)	
		Minimum one seizure recorded	Multiple seizures recorded
Ischemic stroke	75 (27.2%)	22 (29.3%)	17 (22.7%)
IPH	59 (21.3%)	13 (22.0%)	8 (13.6%)
SAH	83 (30.1%)	19 (22.9%)	15 (18.1%)
TBI	53 (19.2%)	9 (17.0%)	6 (11.3%)
Other	6 (2.2%)	1 (16.7%)	0 (0.0%)
Total	276 (100%)	64 (23.2%)	46 (16.7%)

Table 1 shows an even distribution of indications for craniectomy among the cohort of 276 patients who underwent cranioplasty. Numbers and proportions of patients with new-onset seizures are given (single and multiple seizures). Differences in the rate of new-onset seizures after cranioplasty between DHC indication groups were not significant. *CP*, cranioplasty; *DHC*, decompressive hemicraniectomy; *IPH*, intraparenchymal hemorrhage; *SAH*, subarachnoid hemorrhage; *TBI*, traumatic brain injury

### New-onset seizures after cranioplasty

In total, 88 of the 276 patients (31.9%) suffered from seizures in the course of their disease. In 24 patients (8.7%), however, the first seizure occurred already between DHC and CP. In the remaining 64 patients (23.2%), the first seizure occurred after CP; hence, those cases were classified as NOSAC. Of these 64 patients who suffered from new-onset seizures, in 46 (71.9%) multiple seizures were documented.

### Presumed risk factors and time of follow-up

**Fig. 1:**
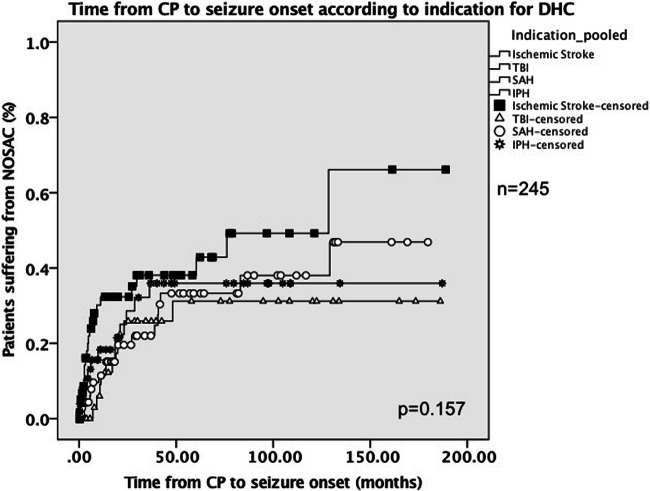
Time from CP to seizure onset according to indication for DHC. Figure 1 shows the time to seizure onset for each patient group according to indication for DHC. Four patients with other indications than those listed above were excluded from this Kaplan-Meier plot due to low case number. Furthermore, one patient was excluded due to very long time between CP and seizure onset (2926 days) to enable appropriate visualization. The differences in time to seizure onset between the groups were not significant according to log-rank test. CP, cranioplasty; DHC, decompressive hemicraniectomy; IPH, intraparenchymal hemorrhage; SAH, subarachnoid hemorrhage; TBI, traumatic brain injury

The rate of patients suffering from NOSAC was independent of the type of underlying pathology (Tables 1 and 2). Timing of cranioplasty, patient age, presence of a VP shunt, use of artificial material for CP, and incidence of postoperative complications had no effect on the rate of new-onset seizures. Actuarial rates of patients suffering from NOSAC are visualized via the Kaplan-Meier plot in Fig. 1. Only time of follow-up was significantly associated with the incidence of new-onset seizures after CP. The median time of follow-up was 58 months (0–189 months) in patients with NOSAC and 19 months (0–186 months) in patients without NOSAC (*P* < 0.001). Furthermore, logistic regression confirmed this finding, as it showed that the incidence of seizures was dependent on the time of follow-up (*P* < 0.001). As depicted in Table 2, eight patients with NOSAC additionally suffered from postoperative complications including subdural or epidural hematoma, bone flap resorption, or bone flap infection. The median time between postoperative complications and onset of seizures in those patients was 308.5 days (30–1427 days).

**Table 2:** Presumed risk factors for new-onset seizures after CP

Presumed risk factor (*n* = 252)	Patients with new-onset seizures after CP (*n* = 64)	Patients w/o new-onset seizures after CP (*n* = 188)	*P* value (chi^2^/logistic regression)
Indication for DHC	see Table 1	see Table 1	0.515
Artificial material used for CP (%)	4 (6.3%)	10 (5.3%)	0.757
VP shunt prior to CP (%)	14 (21.9%)	35 (18.6%)	0586
Postoperative complications after CP (%)	8 (12.5%)	36 (19.1%)	0.258
Median age at CP in years (min–max)	49 (19–75)	50 (18–81)	0.828
Median time between DHC and CP in days (min–max)	103 (13–275)	89 (11–2926*)	0.287
Median time of follow-up in months (min–max)	58 (0–189)	19 (0–186)	< 0.001

Table 2 shows presumed risk factors for new-onset seizures after CP according to current literature and the association of the incidence of new-onset seizures with time of follow-up. *In one case, time between DHC and CP was 2926 days; this patient initially refused to undergo cranioplasty for 8 years, until he finally scheduled an appointment at the outpatient clinic. *CP*, cranioplasty; *DHC*, decompressive hemicraniectomy; *VP*, ventriculoperitoneal

### Timing of cranioplasty and seizure onset

The overall median time between DHC and CP was 95 days (8–2918 days). As depicted in Table 3, time between DHC and CP differed significantly between patients grouped according to the indication for DHC. For patients who suffered from NOSAC, the median time between CP and first seizure was 272 days (4–3874 days, *n* = 63, one patient excluded due to insufficient medical record). Only six of the 63 patients (9.5%) suffered from their first seizures within the first month after surgery, whereas in 73.6% (47/63) time between CP and first seizure was minimum 3 months. There was no significant difference in the median time between CP and onset of NOSAC or median time between DHC and onset of NOSAC between indication groups.

**Table 3:** Median time from DHC to CP, from CP to first seizure, and from DHC to first seizure

Indication for DHC (*n* = 64)	Median time from DHC to CP in days (min–max)	Median time from CP to first seizure in days (min–max)	Median time from DHC to first seizure in days (min–max)
Ischemic stroke (*n* = 22)	140 (25–264)	138.5 (13–3857)	315 (106–3967)
IPH (*n* = 13)	95 (13–275)	183 (7–1088)	249 (51–1148)
SAH (*n* = 19)	66 (23–239)	489.5 (4–3874)	561 (79–3981)
TBI (*n* = 9)	66 (22–187)	514 (212–1445)	588 (246–1512)
Other (*n* = 1)	175	267	442
*P* value (Kruskal-Wallis)	**0.002**	0.155	0.461

Table 3 shows that the median time from DHC to CP differed significantly between patients, grouped according to indication for DHC. There was no significant difference, however, in time from CP to first seizure or in time from DHC to first seizure. *CP*, cranioplasty; *DHC*, decompressive hemicraniectomy; *IPH*, intraparenchymal hemorrhage; *SAH*, subarachnoid hemorrhage; *TBI*, traumatic brain injury

The bold value (0.002) is the p-value for Kruskal Wallis test of differences between the median times between DHC and CP according to DHCindication-groups. As mentioned in the "methods" section, p-values blow 0.05 were regarded as significant. Thus the difference between median times between DHC and CP is highliy significant

## Discussion

There are numerous reports about risk factors for new-onset seizures after CP including indication for DHC, timing of CP, patient age at CP, and use of artificial materials for CP. However, those do not seem to show a consistent pattern [13, 16, 18–20]. Rates of new-onset seizures after CP differ substantially between studies and variables identified as risk factors among one cohort do not seem to have any influence in others. We suppose the reason for these conflicting data to be the massive bias based on mainly two assumptions: (1) The underlying pathology that lead to DHC is the main driver in the genesis of new-onset seizures. Consequently, the causative connection of new-onset seizures and the surgical procedure of a cranioplasty cannot be easily derived. In the current study, 24 patients suffered from seizures after DHC and before CP. These 24 patients represent 27.3% of patients who suffered from seizures in this cohort and 8.7% of the overall cohort. (2) There is a considerable inhomogeneity in the time of follow-up between different studies. According to the current study, the time of follow-up is clearly associated with the incidence of NOSAC, which is in line with the common understanding of epileptogenesis in general [3, 8, 12]. Consequently, it seems likely that other factors which potentially influence the time of follow-up, such as timing of CP or postoperative complications, may falsely be identified as risk factors for NOSAC.

### Clinical and biological factors

Several mechanisms that may lead to epileptic seizures after intracranial hemorrhage, ischemic stroke, and traumatic brain injury have been proposed. However, the exact etiology remains unclear. In patients suffering from post-ischemic seizures, increase of glutamate, destruction of cell membranes, disturbance of electrolyte balance, and secretion of free fatty acids are thought to be crucial [23]. Numerous reports about post-ischemic strokes exist and rates of new-onset seizures after ischemic stroke up to 50% have been reported [2, 7, 14]. In cases of intracranial hemorrhage, seizures are thought to be caused by products of blood metabolism [23]. Consequently, Jo et al. reported that nanoscale iron injection into the adult mouse cortex leads to a reduction in the number of GABAergic interneurons [6]. The rate of seizures after intracranial hemorrhage has been reported to be around 10–15% for intracerebral hemorrhage and 10–20% for subarachnoid hemorrhage, respectively [1, 4, 5]. In cases of traumatic brain injury, mechanisms including neuroinflammation, tauopathy, and mediation by toll-like receptors are discussed to cause seizures in 10–20% of patients [10]. Although contusion and/or hemorrhage may occur during the procedure of cranioplasty, there does not seem to be a relation between the occurrence of post- or intraoperative complications and the incidence of NOSAC according to the current study. To the best of our knowledge, other mechanisms that could lead to the occurrence of seizures after CP have not been reported so far.

### Timing of cranioplasty

According to numerous reports, timing of CP is a significant driver for the development of new-onset seizures. While many studies showed an association of late CP with NOSAC, others reported the opposite [13, 16, 18, 20]. In a systematic review by Malcolm et al. on the other hand, no effect of timing of CP could be observed [11]. We suggest the following explanatory model for these contradictory results: Given the fact that the initial disease that led to DHC is the main cause of new-onset seizures in the majority of patients, timing of CP only resembles a point in time according to which seizures will later be determined as NOSAC or classified as pre-existing seizures. Consequently, patients undergoing late cranioplasty will be more likely classified into the non-new-onset seizure group. As patients with seizures prior to CP are excluded when determined new-onset seizures after CP, more patients with early CP will remain in the study cohort. According to the initial proportions of early and late cranioplasty as well as to individual policies of timing of CP, it seems likely then that the evaluation of timing of CP as a driving factor results in an apparent association between timing of CP and new-onset seizures. In the current study, however, timing of CP was not associated with the incidence of new-onset seizures. As depicted in Table 3, there was a significant difference in the median time from DHC to CP between indication groups, but no significant difference could be observed in the median time from DHC to first seizure or CP to first seizure. These results would rather suggest that new-onset seizures are independent of the procedure of CP and are more likely to be caused by the initial disease, again supporting the common understanding of epileptogenesis.

### Time of follow-up

Time of follow-up seems to be a crucial factor for the evaluation of NOSAC. In the current study, the median time of follow-up in patients with NOSAC was three times longer than of patients without NOSAC (59 vs 19 months). Furthermore, the median time between CP and the first seizure in the current study was 272 days (4–3874 days) suggesting that a considerable proportion of late-onset seizures will not be observed in cohorts without a sufficient proportion of patients with long-term follow-up. According to a systematic review by Spencer et al., many studies focusing on NOSAC lack a sufficient follow-up time, which might contribute to the considerable bias [18]. This hypothesis is in line with the data of previous reports: Morton et al. report 4.1% NOSAC with a median follow-up of 0.6 years, Lee et al. report 14.81% NOSAC with a minimum follow-up of 1 year, Yeap et al. report 26.5% NOSAC with a minimum follow-up of 2 years, and Shih et al. report 30.3% NOSAC with a median follow-up of 2 years [9, 13, 16, 21]. According to individual possibilities and policies of each institution, time of follow-up might depend on timing of CP, which again would contribute to falsely address timing of CP being interpreted as a risk factor for seizures.

## Limitations

Our study is limited due its retrospective character. Furthermore, individual policies concerning timing of CP as well as indications for DHC within a patient cohort may vary considerably between different centers. Our hypothesis can only be underpinned by the data presented; to prove it, however, a prospective study with a high case number would be needed.

## Conclusion

New-onset seizures after cranioplasty may be a direct consequence of patients’ initial condition leading to decompressive hemicraniectomy and may be independent of the cranioplasty procedure itself or at least much less dependent of the procedure than suggested in previous reports. While the actual impact of the reported risk factors remains unclear, the time of follow-up seems to be the main driver in the observed incidence of new-onset seizures after cranioplasty.
